# Supplementary material for: Evolutionary assembly of a unique purple-green photosymbiosis revealed by expanded ciliate diversity
Source: ISME J. 2026 Jun 7;20(1):wrag142. doi: 10.1093/ismejo/wrag142 (PMC13310139; doi:10.1093/ismejo/wrag142)
Supplement: Supplementary_material_wrag142 [file supplementary_material_wrag142.zip › SupplementaryFigureLegends_2026_-_05_-_28_wrag142.docx]

**Supplementary figures legends**

**Figure S1. Phylogeny of the Spirostomidae based on the rRNA gene operon and mitochondrial COI gene.** The tree is rooted between the genus *Anigsteinia* and all other taxa. See Methods for details. Support values are SH-aLRT (%) / ultrafast bootstrap (%) / non-parametric bootstrap (%). Black circles represent branch support values of ≥90% / ≥90% / ≥90%. Gray circles represent branch support values of ≥70% / ≥70% / ≥70%. White circles represent branch support values that do not meet either of these criteria.

**Figure S2. Phylogeny of the Spirostomidae based on 203 proteins and the LG4X model.** The tree is rooted on a selection of non-spirostomid heterotrichids. Support values are SH-aLRT (%) / ultrafast bootstrap (%). Black circles represent branch support values of ≥90% / ≥90%. Gray circles represent branch support values of ≥70% / ≥70%. White circles represent branch support values that do not meet either of these criteria.

**Figure S3. Phylogeny of the Spirostomidae based on 203 proteins and the LG+C60+G4+F model.** The tree is rooted on a selection of non-spirostomid heterotrichids. Support values are SH-aLRT (%) / ultrafast bootstrap (%). Black circles represent branch support values of ≥90% / ≥90%. Gray circles represent branch support values of ≥70% / ≥70%. White circles represent branch support values that do not meet either of these criteria.

**Figure S4. Phylogeny of the Spirostomidae based on 203 proteins and the LG+PMSF(C60)+G4+F model.** The tree is rooted on a selection of non-spirostomid heterotrichids. Support values are SH-aLRT (%) / ultrafast bootstrap (%). Black circles represent branch support values of ≥90% / ≥90%. Gray circles represent branch support values of ≥70% / ≥70%. White circles represent branch support values that do not meet either of these criteria.

**Figure S5. Micrograph plate of *Pseudoblepharisma* sp. PsK1 (Ulsan, South Korea). A.** Whole cell body view of specimen 1. **B.** Whole cell body view of specimen 2. **C.** Closeup of peristome, and adoral membrane (am), and cytostome. **D.** Close up view of ciliary rows and cortical granules. **E.** Close up view of ciliary rows. **F.** Oval macronucleus (ma). **G.** Posterior contractile vacuole (cv) and digestive vacuoles (arrowheads). Scale bars: 55 µm (A, B), 10 µm (C, D, E, F, G).

**Figure S6. Summary of the metagenome of *P. chlorelligerum*.** Anvi‘o diagram of the metagenome of the *P. chlorelligerum* symbiotic consortium. Scaffolds are organized according to their tetranucleotide composition. The coverage values correspond to four short-read Illumina libraries (WGA3, WGA4, WGA5, and WGA6) made from three different *P. chlorelligerum* single cells. The colored taxonomy ticks correspond to contigs whose genes are labeled “Ciliophora”, “Chlorophyta”, and “Candidatus Accumulibacter” and “Propionivibrio” based on similarity searches.

**Figure S7. Comparison of the presence/absence of core metabolic functions between the bacterial endosymbionts *P. chlorelligerum* and their relatives.** In the grid, each column is a core metabolic function with the gene names provided as the column name and each row is a species in the *Rhodocyclaceae*. The dark purple shaded boxes depict the presence of a function in the genome, while white/light purple depict absence. The *Accumulibacter* and *Propionivibrio* symbionts of *P. chlorelligerum* are highlighted in light purple. The functions are grouped into metabolic categories that are highlighted in different colors at the top of the columns. The phylogenetic relationships of the species are shown to the right of the grid.

**Figure S8. Comparison of the key steps in amino acid metabolism across *P. chlorelligerum* and its bacterial endosymbionts.** The amino acid metabolic pathways are listed across the x-axis and each KEGG metabolic step per pathway on the y-axis.

**Figure S9. KEGG map of nitrogen metabolism across *P. chlorelligerum* and its bacterial endosymbionts.** The gene product boxes are colored purple if present in the *Propionivibrio* symbiont, green if present in the *Accumulibacter* symbiont, and orange if present in *P. chlorelligerum* host*.*

**Figure S10. Comparison of the key steps in cofactor and vitamin synthesis across *P. chlorelligerum* and its bacterial endosymbionts.** The cofactor and vitamin metabolic pathways are listed across the x-axis and each KEGG metabolic step per pathway on the y-axis.

**Figure S11. KEGG map of porphyrin metabolism across *P. chlorelligerum* and its bacterial endosymbionts.** The gene product boxes are colored purple if present in the *Propionivibrio* symbiont, green if present in the *Accumulibacter* symbiont, and orange if present in *P. chlorelligerum* host*.*

**Figure S12. Evidence of PHB granules in the *Accumulibacter* symbiont.** Nile Blue A staining *Accumulibacter* symbionts released from the cytoplasm of *P. chlorelligerum*. Arrowheads point to fluorescently labeled PHB granules. See Methods for details. Scale bar: 5 µm.

**Figure S13. Phylogeny of the Chlorellaceae based on the 18S and 5.8S rRNA genes and ITS1 and ITS2 regions.** The tree is rooted in between *Parachlorella* clade and all other taxa. See Methods for details. Support values are SH-aLRT (%) / ultrafast bootstrap (%) / non-parametric bootstrap (%). Black circles represent branch support values of ≥90% / ≥90% / ≥90%. Gray circles represent branch support values of ≥70% / ≥70% / ≥70%. White circles represent branch support values that do not meet either of these criteria.

**Figure S14. Phylogeny of the Chlorellaceae based on the chloroplast *rbcL* gene.** The tree is rooted in its midpoint. See Methods for details. Support values are SH-aLRT (%) / ultrafast bootstrap (%) / non-parametric bootstrap (%). Black circles represent branch support values of ≥90% / ≥90% / ≥90%. Gray circles represent branch support values of ≥70% / ≥70% / ≥70%. White circles represent branch support values that do not meet either of these criteria.

**Figure S15. Phylogenetic distribution of anaerobic energy metabolic enzymes in representative species of the Ciliophora.** Candidate homologs were identified based on reciprocal best-hit (RBH) similarity searches. Dark grey corresponds to detected RBHs and white to absence. Light grey corresponds to conserved complexes that are inferred to be present but had no RBH most likely due to incomplete transcriptome/genome data. Red corresponds to large phylogenetic blocks of absent RBHs.

**Supplementary tables**

**Table S1. *Spirostomum-* and *Pseudoblepharisma*-like cells sampled in this study.**

**Table S2. Single-cell genome and transcriptome data generated as part of this study.** *Species that do not have an associated 18S rRNA gene in Table S1.

**Table S3. Mean coverage values for each symbiont genome bin across six different sequenced WGA libraries.**

**Table S4. Functional annotations of the predicted proteins of *P. chlorelligerum* and its bacterial endosymbionts.**

**Table S5. Predicted KEGG metabolic functions of *Accumulibacter* and *Propionivibrio.* symbionts and their relatives.** This table is the result of a METABOLIC analysis which is itself the basis for Fig. S7, S8 and S10.
