# Supplementary material for: Evolutionary assembly of a unique purple-green photosymbiosis revealed by expanded ciliate diversity
Source: ISME J. 2026 Jun 7;20(1):wrag142. doi: 10.1093/ismejo/wrag142 (PMC13310139; doi:10.1093/ismejo/wrag142)

Supplementary Figure 1

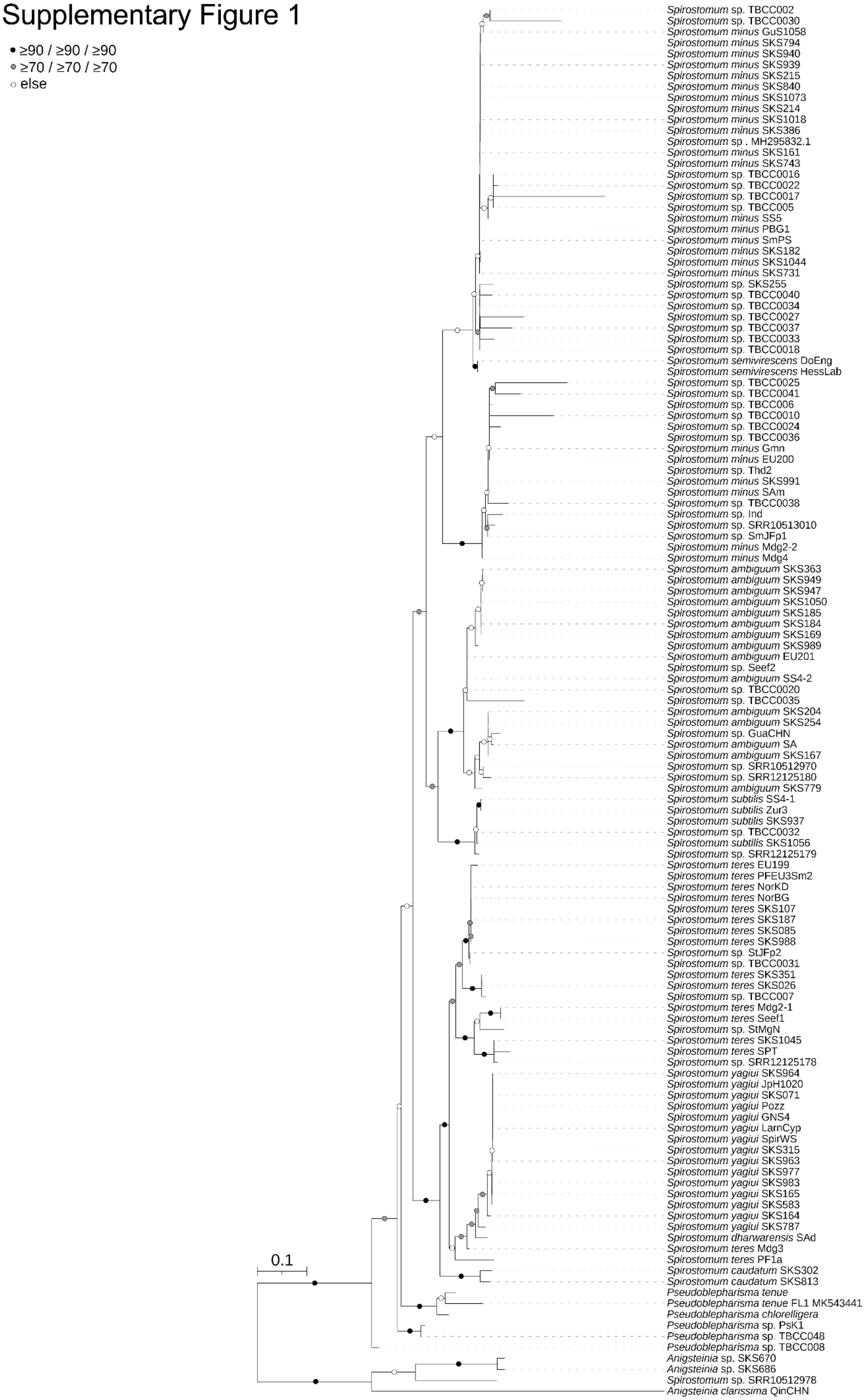

Supplementary Figure 2

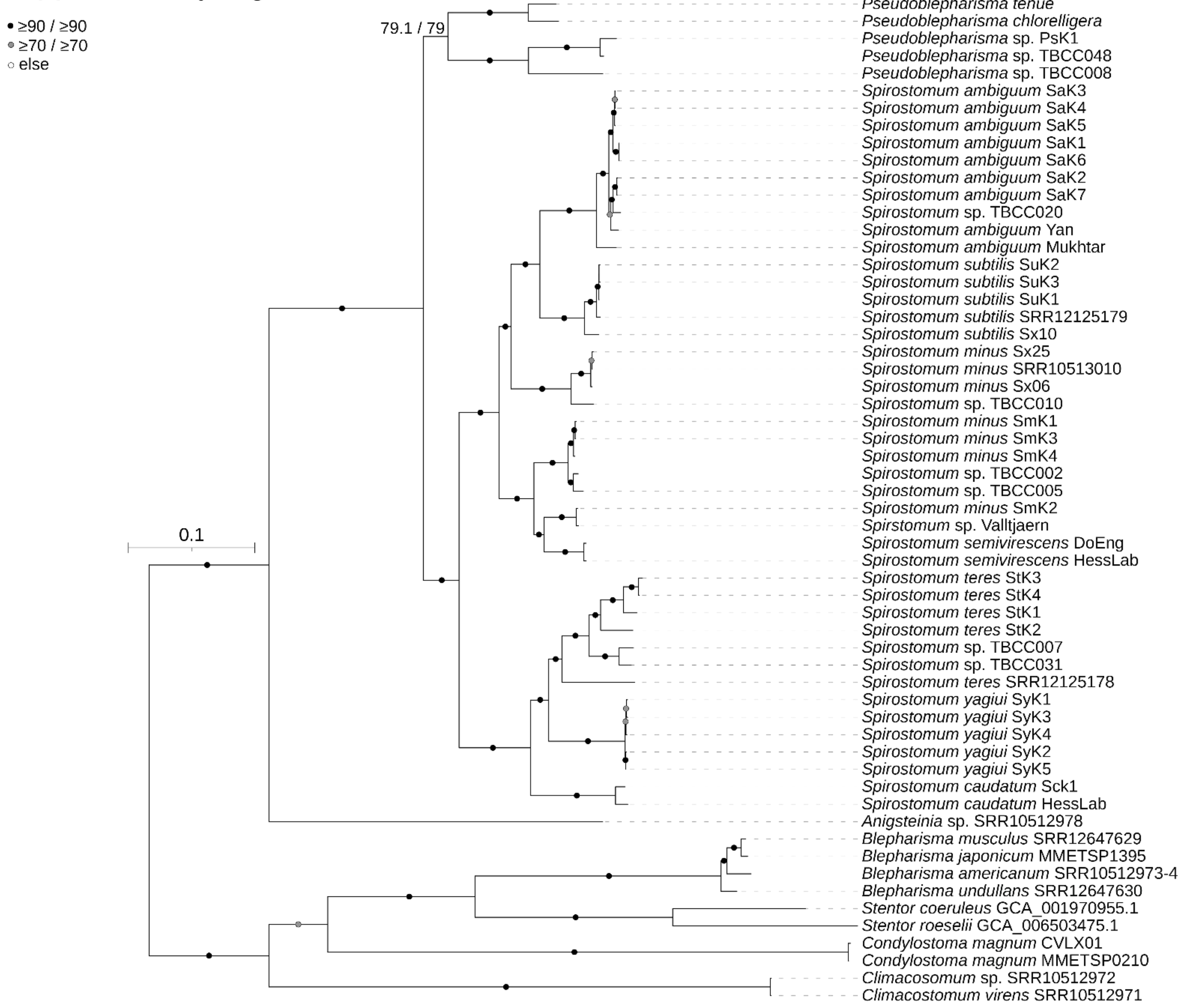

Supplementary Figure 3

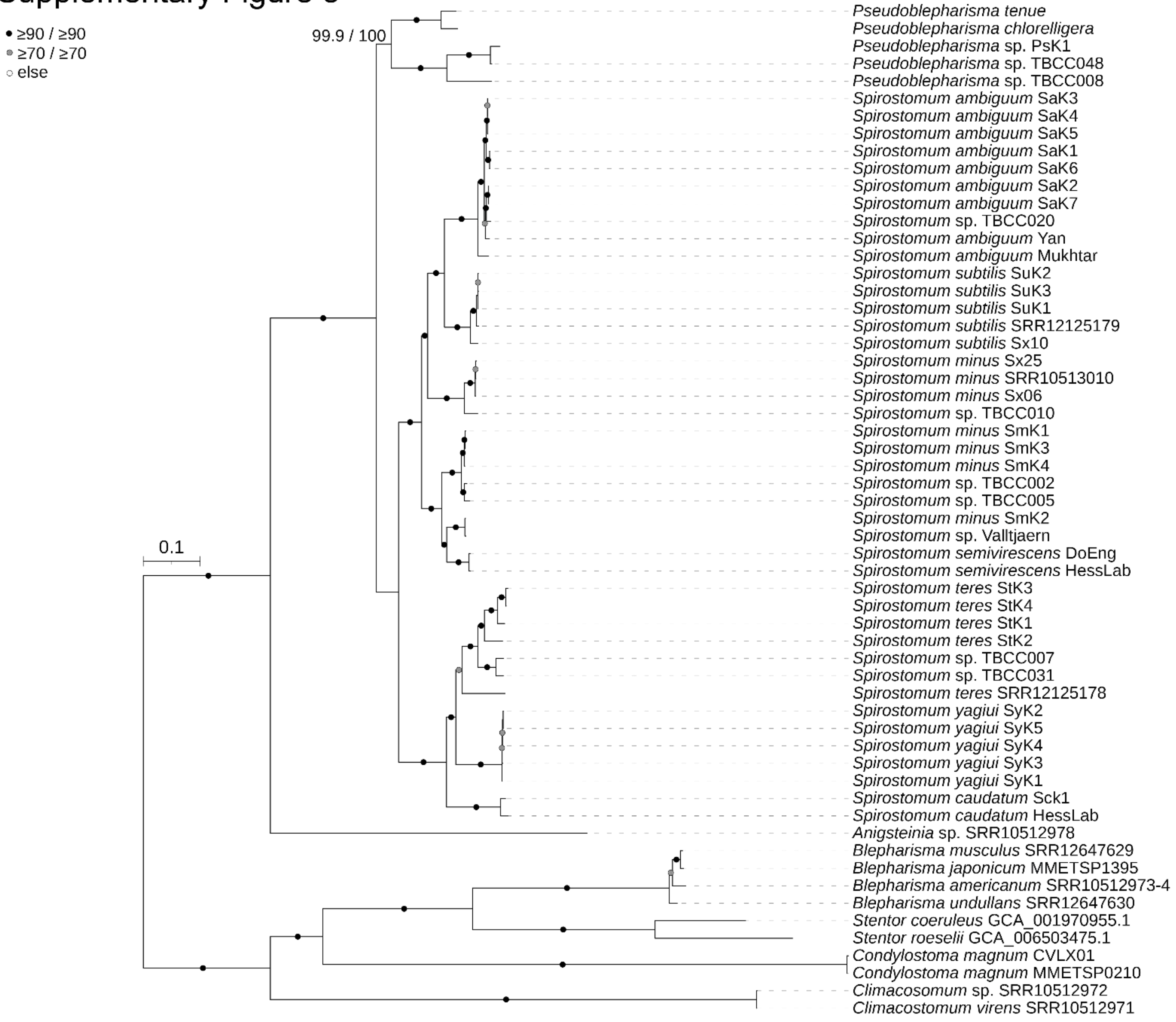

Supplementary Figure 4

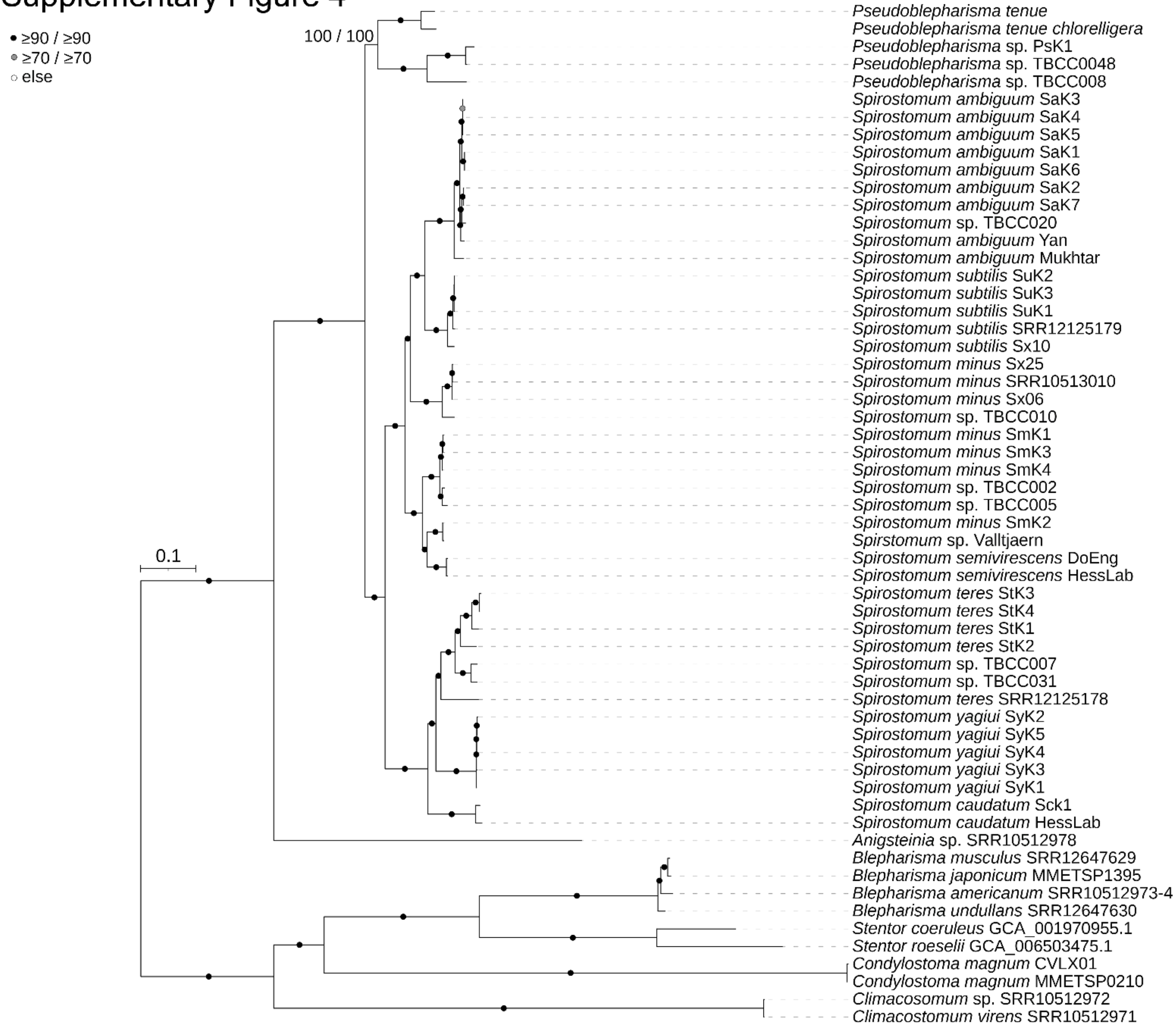

Supplementary Figure 5

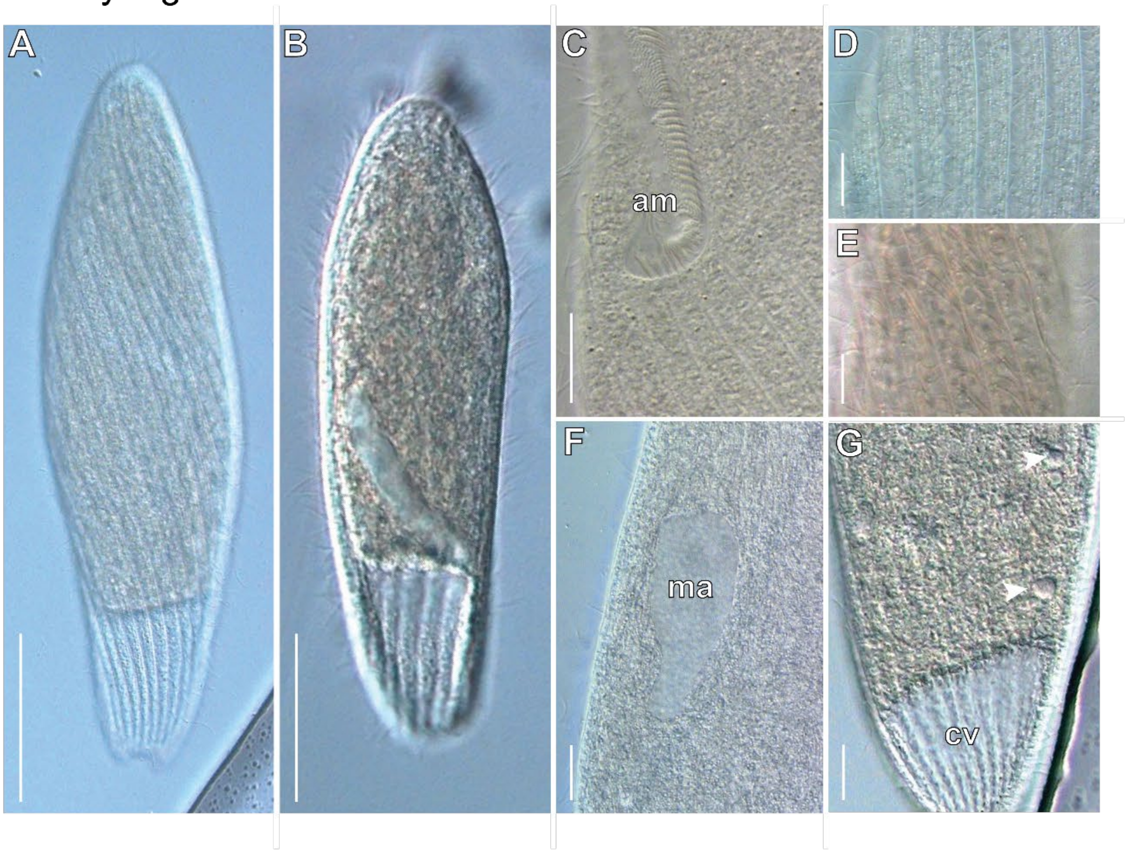

Supplementary Figure 6

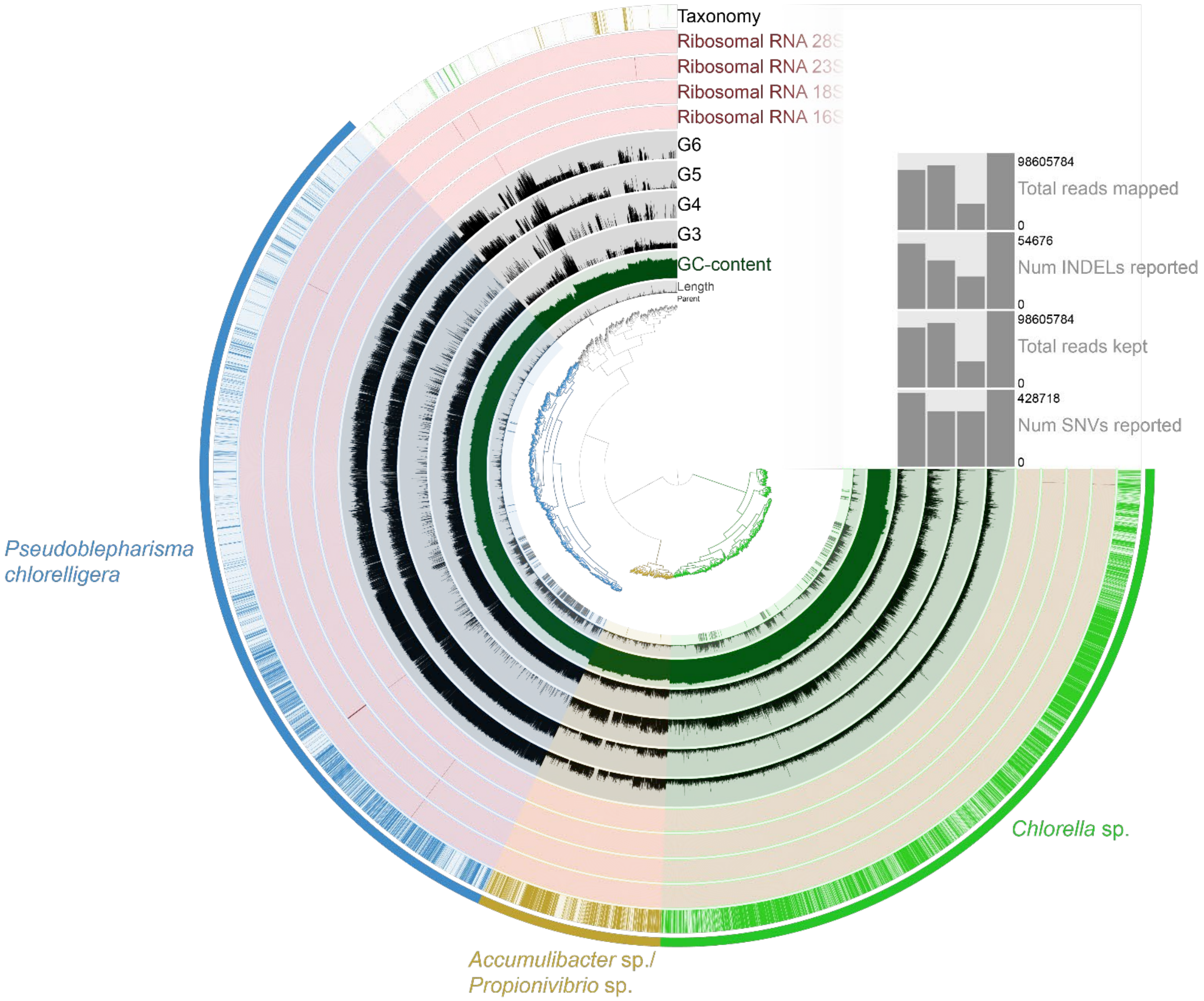

Supplementary Figure 7

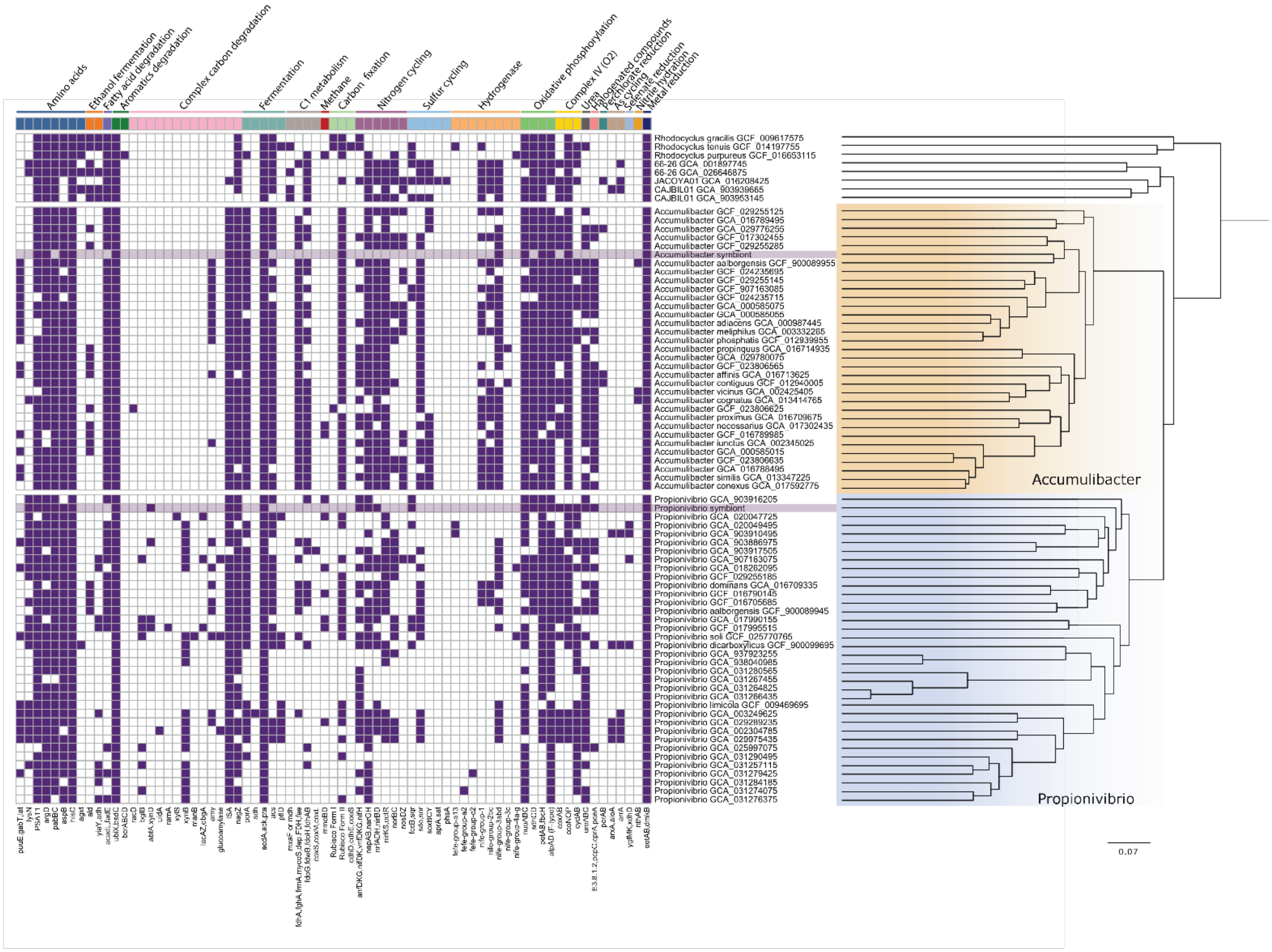

Supplementary Figure 8

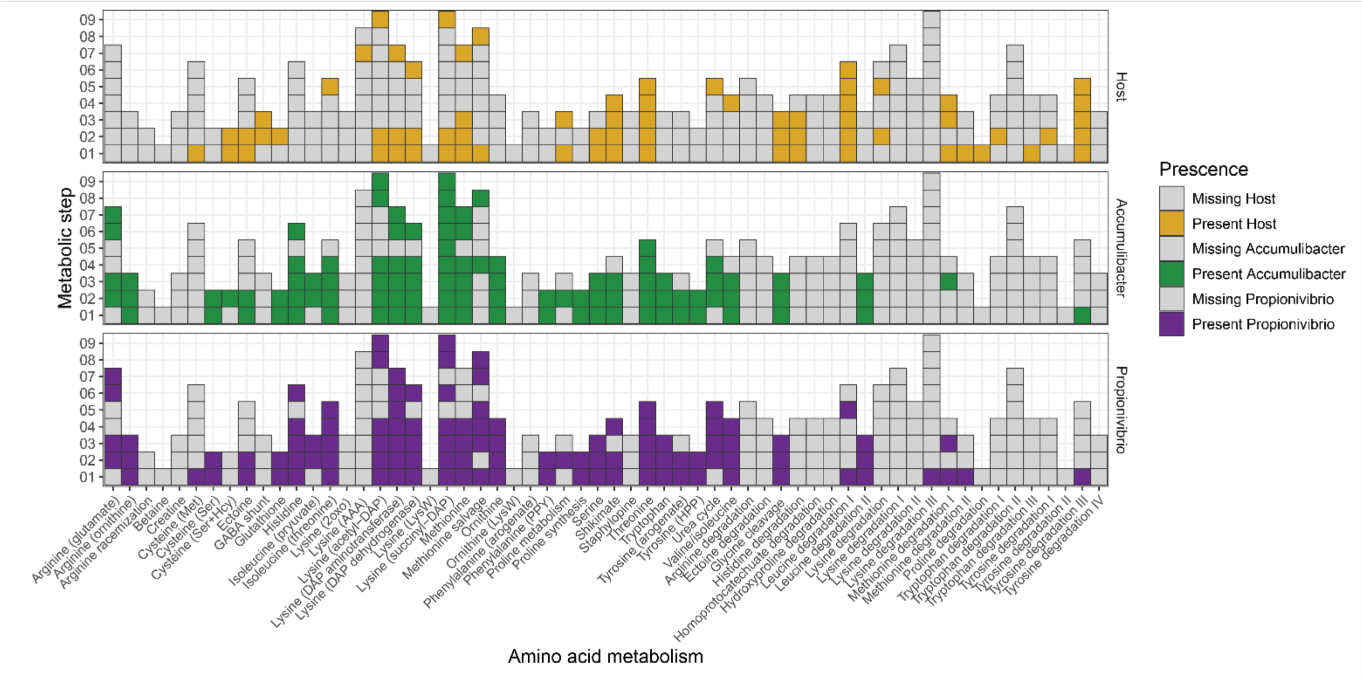

Supplementary Figure 9

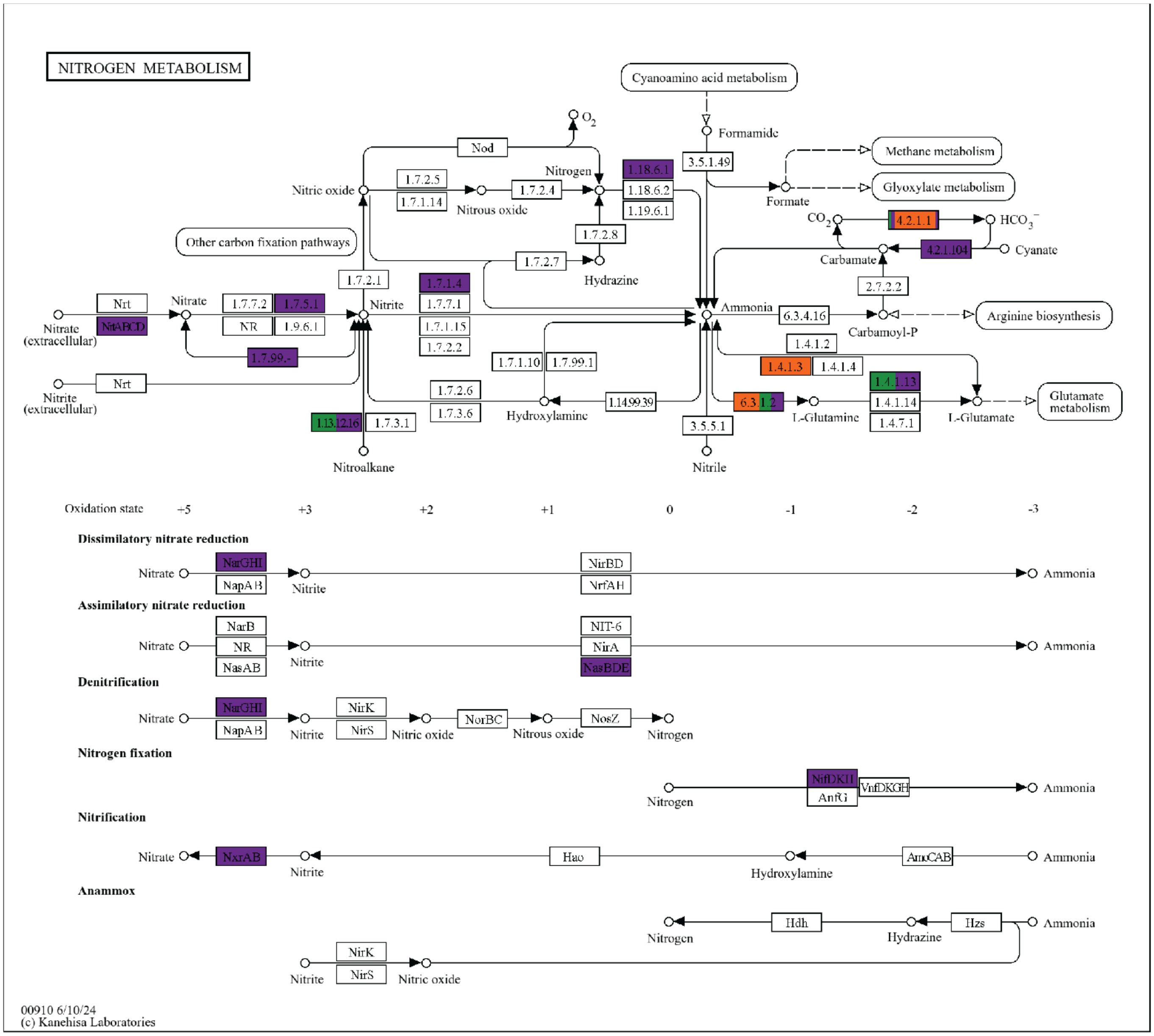

Supplementary Figure 10

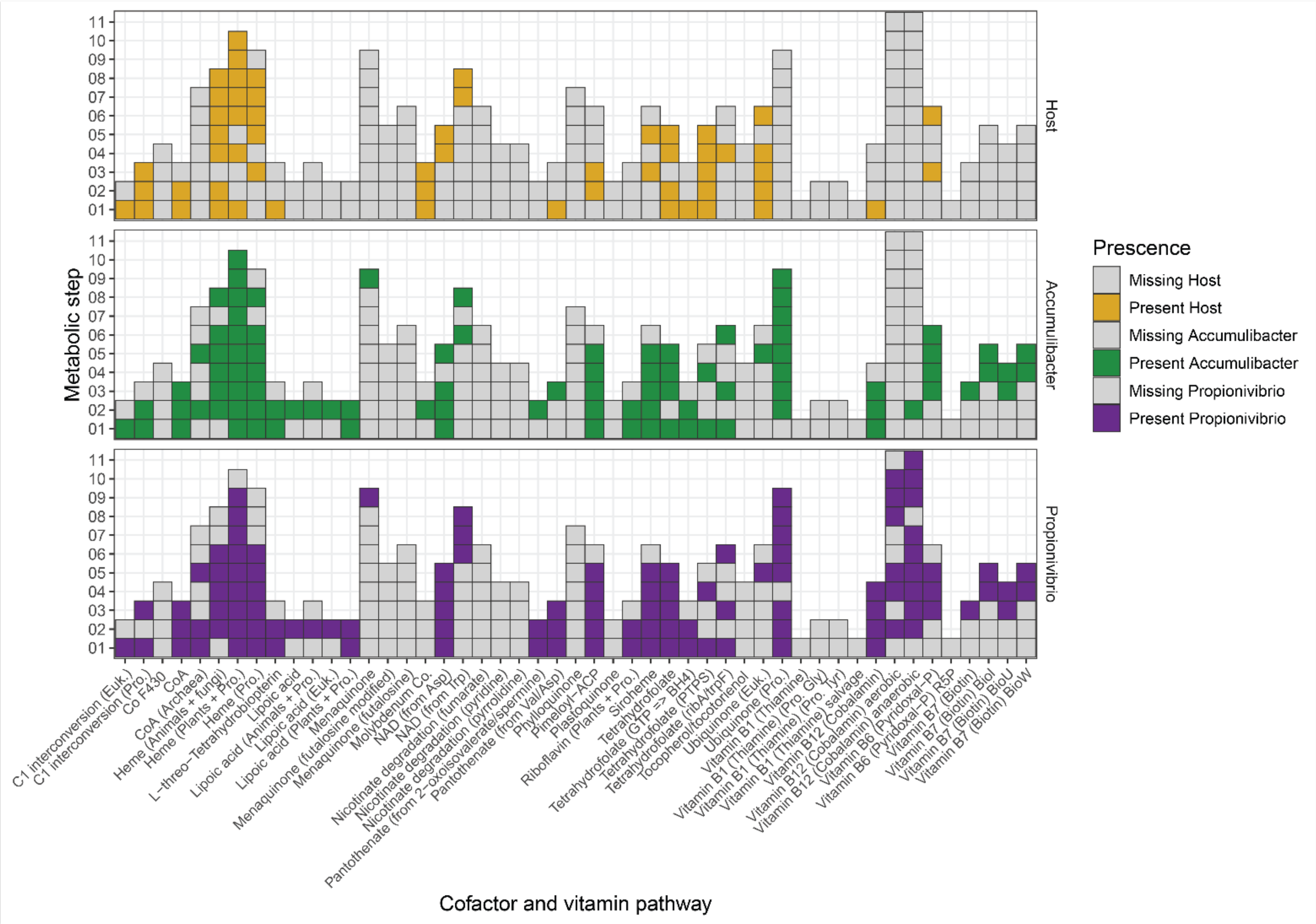

Supplementary Figure 11

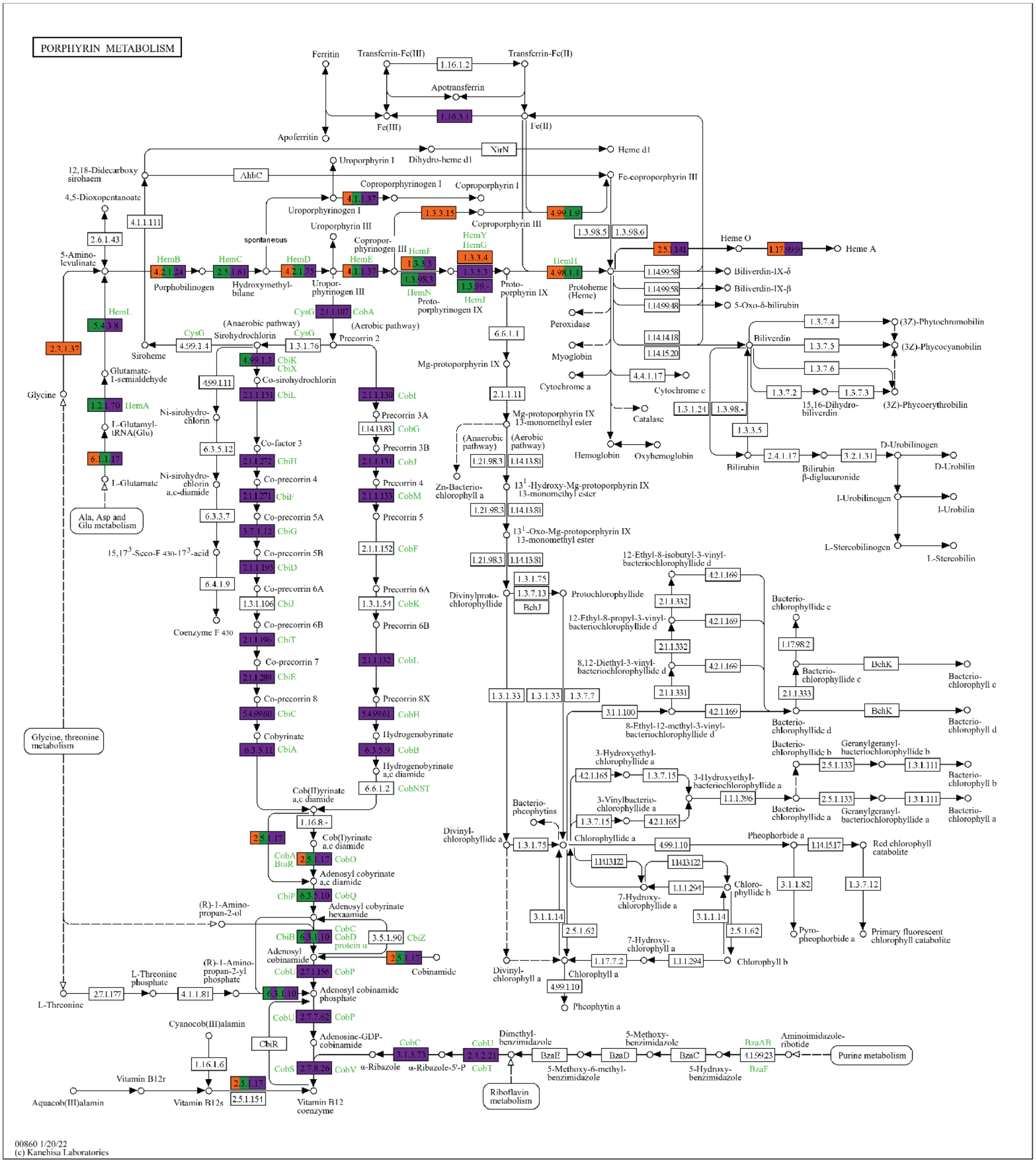

Supplementary Figure 12

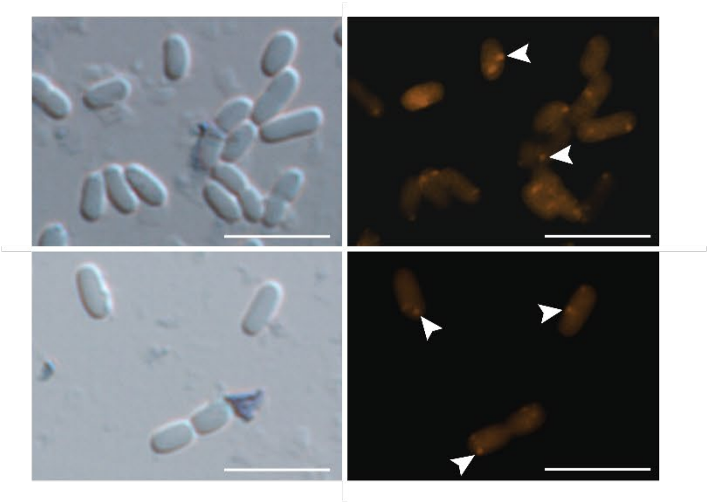

Supplementary Figure 13

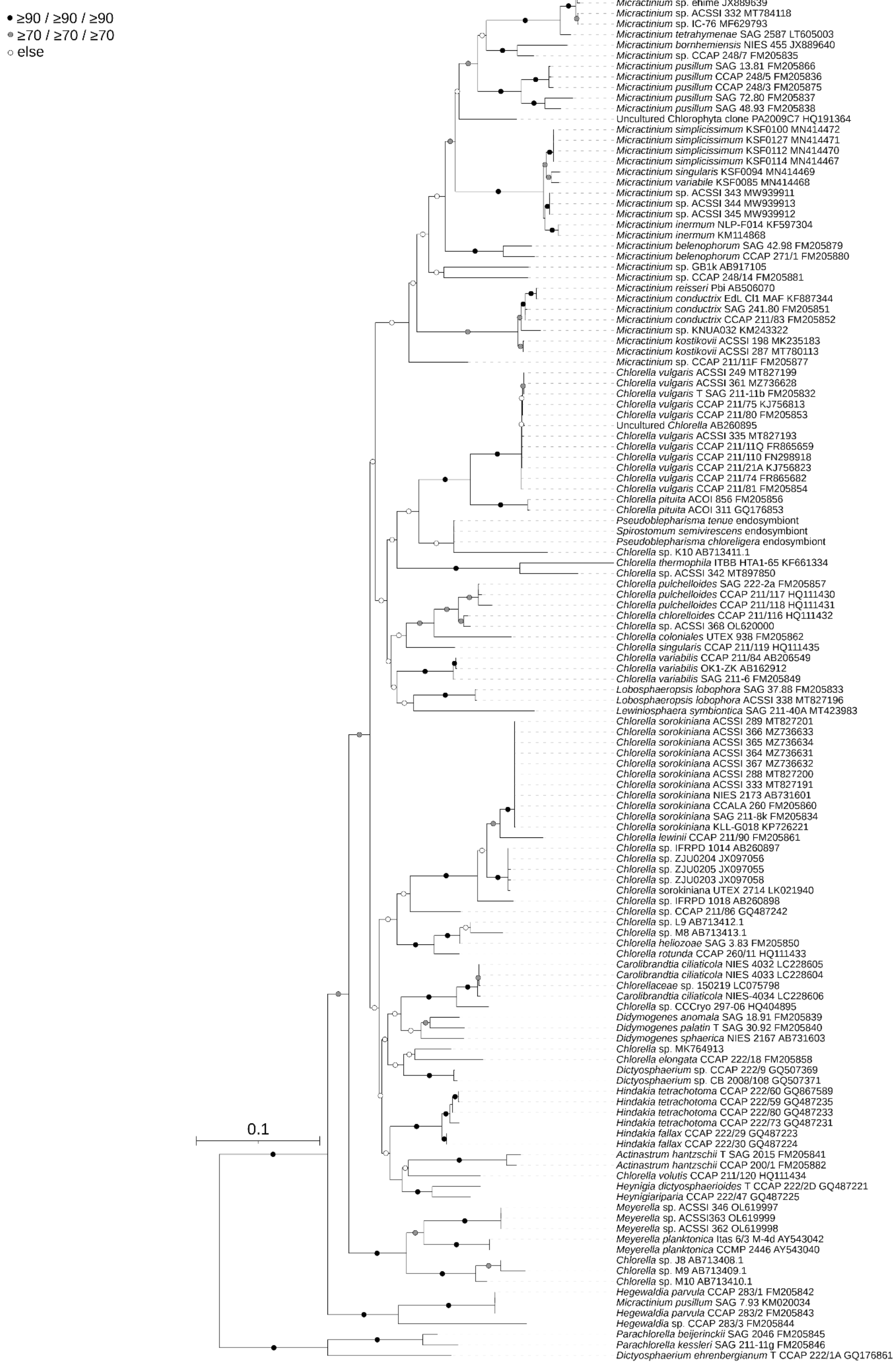

Supplementary Figure 14

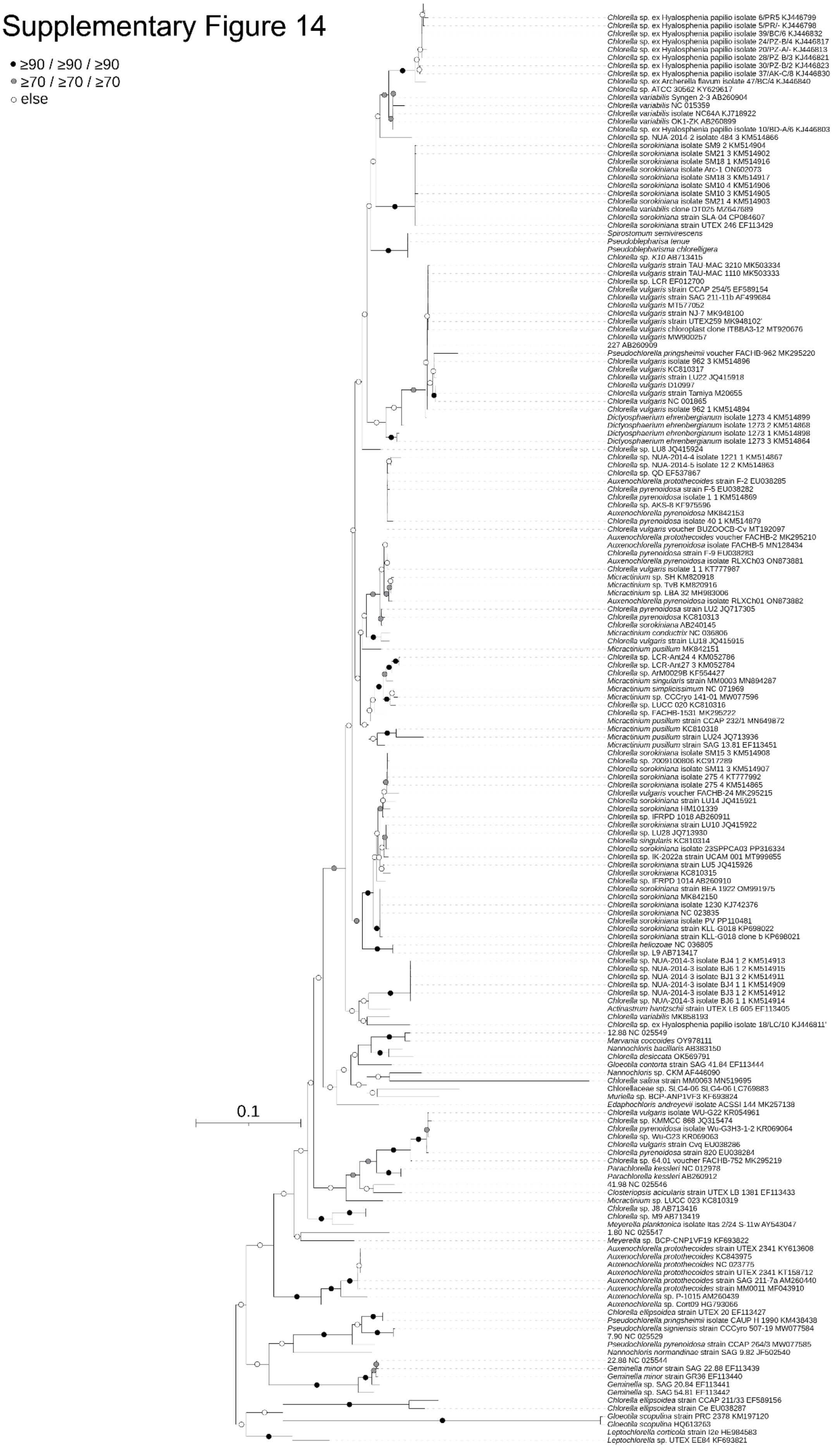

Supplementary Figure 15

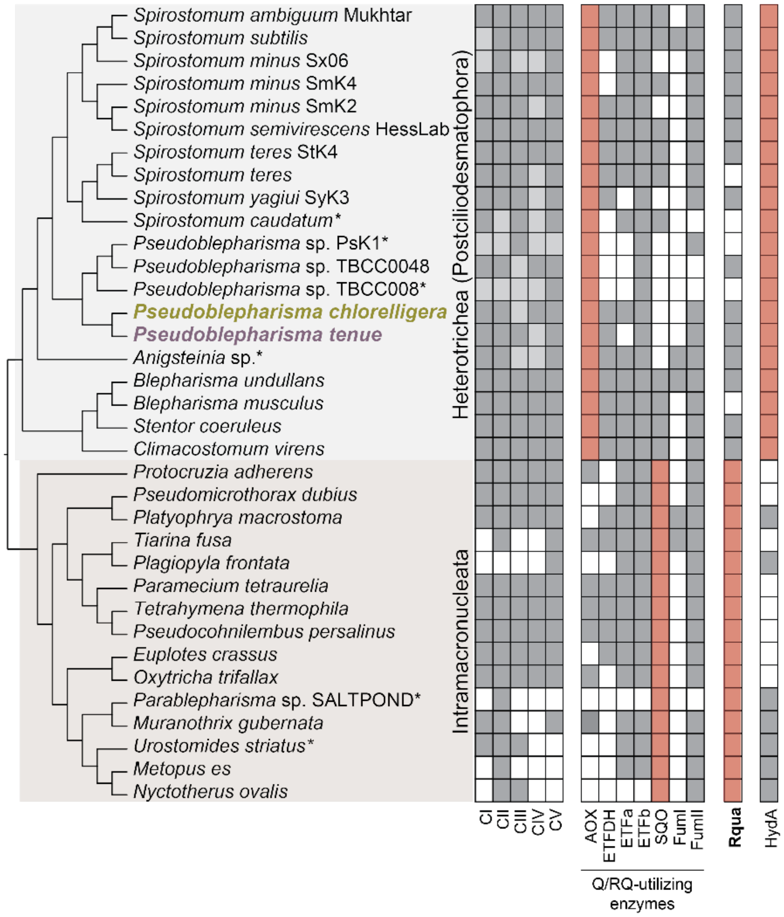

Supplement: Supplementary_material_wrag142 [file supplementary_material_wrag142.zip › SupplementaryFigures_2026_04_-_30_wrag142.pdf]
