# Supplementary material for: Evolutionary assembly of a unique purple-green photosymbiosis revealed by expanded ciliate diversity
Source: ISME J. 2026 Jun 7;20(1):wrag142. doi: 10.1093/ismejo/wrag142 (PMC13310139; doi:10.1093/ismejo/wrag142)
Supplement: Supplementary_material_wrag142 [file supplementary_material_wrag142.zip › Table_S1_wrag142.docx]

**Table S1. *Spirostomum-* and *Pseudoblepharisma*-like cells sampled in this study.**

| **Strain** | **Location** | **Accession number (18S rRNA gene)** |
| --- | --- | --- |
| *Pseudoblepharisma chlorelligerum* | Simmelried, Germany | PZ207284 |
| *Pseudoblepharisma* sp. PsK1 | Ulsan, South Korea | PZ207285 |
| *Pseudoblepharisma* sp. TBCC008 | Rheinbach, Germany | PZ207286 |
| *Pseudoblepharisma* sp. TBCC048 | Regenstauf, Germany | PZ207287 |
| *Spirostomum semivirescens* HessLab | Simmelried, Germany | PZ207289 |
| *Spirostomum caudatum* HessLab | Simmelried, Germany | PZ207288 |
| *Spirostomum* sp. TBCC002 | Kassel, Germany | PZ207294 |
| *Spirostomum* sp. TBCC005 | Heimerzheim, Germany | PZ207311 |
| *Spirostomum* sp. TBCC006 | Huerth, Germany | PZ207312 |
| *Spirostomum* sp. TBCC007 | Huerth, Germany | PZ207313 |
| *Spirostomum* sp. TBCC0010 | Bruehl, Germany | PZ207290 |
| *Spirostomum* sp. TBCC0016 | Heimerzheim, Germany | PZ207291 |
| *Spirostomum* sp. TBCC0017 | Heimerzheim, Germany | PZ207292 |
| *Spirostomum* sp. TBCC0018 | Heimerzheim, Germany | PZ207293 |
| *Spirostomum* sp. TBCC0020 | Heimerzheim, Germany | PZ207295 |
| *Spirostomum* sp. TBCC0022 | Rheinbach, Germany | PZ207296 |
| *Spirostomum* sp. TBCC0024 | Bruehl, Germany | PZ207297 |
| *Spirostomum* sp. TBCC0025 | Huerth, Germany | PZ207298 |
| *Spirostomum* sp. TBCC0027 | Swisttal, Germany | PZ207299 |
| *Spirostomum* sp. TBCC0030 | Heimerzheim, Germany | PZ207300 |
| *Spirostomum* sp. TBCC0031 | Hannover, Germany | PZ207301 |
| *Spirostomum* sp. TBCC0032 | Hannover, Germany | PZ207302 |
| *Spirostomum* sp. TBCC0033 | Heimerzheim, Germany | PZ207303 |
| *Spirostomum* sp. TBCC0034 | Heimerzheim, Germany | PZ207304 |
| *Spirostomum* sp. TBCC0035 | Rheinbach, Germany | PZ207305 |
| *Spirostomum* sp. TBCC0036 | Hannover, Germany | PZ207306 |
| *Spirostomum* sp. TBCC0037 | Simmelried, Germany | PZ207307 |
| *Spirostomum* sp. TBCC0038 | Germany | PZ207308 |
| *Spirostomum* sp. TBCC0040 | Swisttal, Germany | PZ207309 |
| *Spirostomum* sp. TBCC0041 | Bruehl, Germany | PZ207310 |
