# Supplementary material for: Evolutionary assembly of a unique purple-green photosymbiosis revealed by expanded ciliate diversity
Source: ISME J. 2026 Jun 7;20(1):wrag142. doi: 10.1093/ismejo/wrag142 (PMC13310139; doi:10.1093/ismejo/wrag142)
Supplement: Supplementary_material_wrag142 [file supplementary_material_wrag142.zip › Table_S2_wrag142.docx]

**Table S2. Single-cell genome and transcriptome data generated as part of this study.** *Species that do not have an associated 18S rRNA gene in Table S1.

| **Species name** | **Location** | **Type of library** | **Accession number** | **Sequencing technology** | **Size (GB)** |
| --- | --- | --- | --- | --- | --- |
| *Pseudoblepharisma chlorelligerum* | Simmelried, Germany | WGA | SRR25464810 | Illumina | 30.22 |
| *Pseudoblepharisma chlorelligerum* | Simmelried, Germany | WGA | SRR25464809 | Illumina | 89.20 |
| *Pseudoblepharisma chlorelligerum* | Simmelried, Germany | WGA | SRR25464798 | Illumina | 12.63 |
| *Pseudoblepharisma chlorelligerum* | Simmelried, Germany | WGA | SRR25464794 | Illumina | 73.80 |
| *Pseudoblepharisma chlorelligerum* | Simmelried, Germany | WGA | SRR25464793 | Nanopore | 7.10 |
| *Pseudoblepharisma chlorelligerum* | Simmelried, Germany | WGA | SRR25464792 | Nanopore | 14.52 |
| *Pseudoblepharisma chlorelligerum* | Simmelried, Germany | WTA | SRR25464791 | Illumina | 63.32 |
| *Pseudoblepharisma chlorelligerum* | Simmelried, Germany | WTA | SRR25464790 | Illumina | 19.22 |
| *Pseudoblepharisma chlorelligerum* | Simmelried, Germany | WTA | SRR25464789 | Illumina | 34.05 |
| *Pseudoblepharisma* sp. PsK1 | Ulsan, South Korea | WTA | SRR25464806 | Illumina | 10.79 |
| *Pseudoblepharisma* sp. TBCC008 | Rheinbach, Germany | WTA | SRR25464808 | Illumina | 33.01 |
| *Pseudoblepharisma* sp. TBCC008 | Rheinbach, Germany | WTA | SRR25464788 | Illumina | 50.11 |
| *Pseudoblepharisma* sp. TBCC048 | Regenstauf, Germany | WGA | SRR25464807 | Illumina | 32.69 |
| *Spirostomum semivirescens* HessLab | Hemmingen, Germany | WGA | SRR25464797 | Illumina | 28.84 |
| *Spirostomum caudatum* HessLab* | Simmelried, Germany | WTA | SRR25464799 | Illumina | 28.82 |
| *Spirostomum teres* StK2* | Samcheok, South Korea | WTA | SRR25464796 | Illumina | 18.78 |
| *Spirostomum teres* StK4* | Ulsan, South Korea | WTA | SRR25464795 | Illumina | 51.82 |
| *Spirostomum* sp. TBCC002 | Kassel, Germany | WTA | SRR25464805 | Illumina | 27.70 |
| *Spirostomum* sp. TBCC005 | Heimerzheim, Germany | WTA | SRR25464804 | Illumina | 57.52 |
| *Spirostomum teres* TBCC007 | Cologne (Hürth), Germany | WTA | SRR25464803 | Illumina | 75.12 |
| *Spirostomum* sp. TBCC010 | Brühl, Germany | WTA | SRR25464802 | Illumina | 78.87 |
| *Spirostomum ambiguum* TBCC020 | Heimerzheim, Germany | WTA | SRR25464801 | Illumina | 86.87 |
| *Spirostomum* sp. TBCC031 | Hannover, Germany | WTA | SRR25464800 | Illumina | 42.92 |
