# Supplementary material for: Evolutionary assembly of a unique purple-green photosymbiosis revealed by expanded ciliate diversity
Source: ISME J. 2026 Jun 7;20(1):wrag142. doi: 10.1093/ismejo/wrag142 (PMC13310139; doi:10.1093/ismejo/wrag142)
Supplement: Supplementary_material_wrag142 [file supplementary_material_wrag142.zip › Table_S3_wrag142.docx]

**Table S3. Mean coverage values for each symbiont genome bin across six different sequenced WGA libraries.**

|  | **Completeness (BUSCO/CheckM2)** | **WGA3** | **WGA4** | **WGA5** | **WGA6** | **WGA7** | **WGA8** |
| --- | --- | --- | --- | --- | --- | --- | --- |
| ***Pseudoblepharisma chlorelligerum*** | 87.9% (alveolata_odb12) | 305.45 | 2.34 | 122.83 | 388.32 | 76.18 | 155.82 |
| ***Chlorella* sp. K10** | 81.4% (chlorophyta_odb12) | 8.73 | 104.65 | 2.43 | 6.69 | 3.12 | 4.54 |
| ***“Ca. Accumulibacter*  symbioticus”** | 95.2% (CheckM2) | 16.60 | 206.04 | 8.58 | 32.80 | 25.08 | 38.00 |
| ***“Ca. Propionivibrio*  subcutaneus”** | 98.04% (CheckM2) | 6.97 | 57.58 | 2.36 | 6.68 | 3.65 | 5.25 |
